# Combined Application of Controlled-Release Urea and Common Urea Improves Yield and Nitrogen Use Efficiency in Low-Gluten Wheat

**DOI:** 10.3390/plants15172728

**Published:** 2026-09-06

**Authors:** Di Li, Guanghui Du, Liyang Chen, Jianbo Yang, Guobing Wang, Yuhu Lv, Yao Liu, Xun Li

**Affiliations:** 1 Institute of Geographical Sciences, Henan Academy of Sciences, Zhengzhou 450052, China; 2 Xinyang Academy of Agricultural Sciences, Xinyang 464001, China; 3 State Key Laboratory of Soil and Sustainable Agriculture, Institute of Soil Science, Chinese Academy of Sciences, Nanjing 211135, China

**Keywords:** yield, controlled-release urea, nitrogen uptake, nitrogen use efficiency, grain quality

## Abstract

Optimizing nitrogen (N) management is essential for improving grain yield, quality, and nitrogen use efficiency (NUE) in low-gluten wheat, yet the optimal fertilizer strategy remains unclear. A two-year field experiment was conducted to evaluate the effects of common urea (CU), controlled-release urea (CRU), and their combination on yield, quality, NUE, and soil N content in low-gluten wheat. The results showed that, compared with 100% common urea (CU100), 100% controlled-release urea (CRU100) increased grain yield by 8.51% in 2023 and 14.3% in 2024, while 80% controlled-release urea and 20% common urea (CRU80CU20) increased grain yield by 19.2% in 2023 and 23.3% in 2024. These increases were accompanied by enhanced grain number per spike and dry matter accumulation. CRU-based treatments improved NUE, with the highest NUE observed in the CRU80CU20 treatment. Compared with CU100, CRU80CU20 significantly decreased grain protein content while maintaining high grain yield. Path analysis indicated that CRU was positively associated with dry matter accumulation, which in turn was positively associated with grain yield, whereas N translocation was closely associated with grain protein-related quality. In conclusion, CRU80CU20 provided a favorable combination of high grain yield, high NUE, and lower grain protein content, indicating that it is a promising N management strategy for the production of low-gluten wheat.

## 1. Introduction

Nitrogen (N) is a key nutrient that affects wheat yield components and regulates grain quality, and it plays an essential role in crop growth and grain development [1]. However, the average N use efficiency (NUE) of rice, wheat, and maize is only about 39.0%, resulting in over half of the applied fertilizer N not being absorbed by crops [2]. A large proportion of applied N is lost to the environment through ammonia volatilization, nitrate leaching, and denitrification, which not only wastes fertilizer resources but also increases non-point source pollution and greenhouse gas emissions [3,4]. In addition, common urea (CU) is rapidly hydrolyzed after application and releases N in a short period, which may lead to excessive N supply during early growth stages and insufficient supply during anthesis and grain filling stages [5]. This asynchrony between N supply and crop demand, especially the insufficient N availability during the grain-filling stage, often limits wheat yield stability, particularly for quality-sensitive types like low-gluten wheat.

To address the limitations of rapid N release from CU, controlled-release urea (CRU) has received increasing attention in cereal production, as its ability to regulate N release might improve the matching of N supply with crop demand [6,7]. Studies have shown that CRU can significantly increase grain yield, N uptake, and NUE compared with CU and also decrease N leaching and nitrous oxide emissions [8]. However, the sole application of CRU may lead to insufficient N supply during early growth, especially under low-temperature conditions, which may slow CRU release and limit tillering [7]. Recently, more attention has focused on the combined application of CRU and CU, which might meet the N demand at the seedling stage and maintain N supply during later growth stages [9]. Previous studies have shown that this combination of CRU and CU can further increase yield and NUE while reducing ammonia volatilization [10].

Low-gluten wheat, which is mainly used for biscuits, cakes, and steamed products, requires low grain protein and low wet gluten content and is highly sensitive to N supply [11]. According to the national standard GB/T 17320-2013, the crude protein content (dry basis) of low-gluten wheat is less than 12.5%, the wet gluten content (14% moisture basis) is less than 26%, and the Zeleny sedimentation value is less than 30 mL [12]. Therefore, lower grain protein content is an important quality indicator for low-gluten wheat. Excessive N application often leads to high grain protein content, which decreases flour functionality, dough extensibility, and the textural properties of the resulting products [13]. Therefore, N management for low-gluten wheat needs to ensure sufficient N supply to maintain yield while avoiding excessive N supply that leads to protein accumulation. Many studies have shown that regulating the amount, timing, and release pattern of N is critical for achieving high yield and quality [14,15]. Reducing the total N input and optimizing N release, particularly avoiding excessive N supply after flowering, may help suppress excessive protein accumulation while maintaining or increasing yield [16]. Thus, N management strategies for low-gluten wheat should be based on its specific quality requirements.

Although many studies have examined the role of CRU in improving crop yield and NUE, several limitations remain. First, the transfer of dry matter to grains and the redistribution of N among plant organs are key processes that affect yield and quality [17,18], but research on the relationship among dry matter accumulation, N uptake, and their translocation is limited. Second, the N demand pattern of low-gluten wheat differs from that of other wheat types [19]. Particularly, excessive N supply during the grain filling stage may increase protein content and decrease processing quality [20]. Most studies on CRU focus on strong or medium gluten wheat, with insufficient understanding of its effects on yield and quality in low-gluten wheat [21,22]. Therefore, it is important to develop suitable N management strategies and clarify their effects on yield, N uptake, and NUE for low-gluten wheat.

This study aimed to elucidate the effects of CRU and CU on the growth, yield, NUE, and grain quality of low-gluten wheat. The objectives of the study were to (1) clarify the effects of different N strategies on yield and its components; (2) elucidate the regulation of dry matter accumulation, N uptake, translocation, and their contributions to grain yield formation; and (3) evaluate soil N content and NUE under different N treatments and to identify an optimal N management strategy that achieves high yield, favorable quality, and high NUE. We hypothesized that the combined application of CRU and CU would improve the yield, dry matter accumulation, and NUE of low-gluten wheat while avoiding excessive grain protein accumulation.

## 2. Results

### 2.1. Effects of Combined Application of Controlled-Release Urea and Common Urea on Yield and Yield Components of Low-Gluten Wheat

As shown in Table 1, compared with the treatment without N fertilizer (CK), different N treatments significantly increased plant height, effective spike number, grain number per spike, thousand grain weight, and grain yield in 2023 and 2024. Compared with 100% common urea (CU100) treatment, the 100% controlled-release urea (CRU100) treatment significantly increased grain number per spike and yield by 5.83% and 8.51% in 2023 and by 18.5% and 14.3% in 2024, respectively. The 80% controlled-release urea and 20% common urea (CRU80CU20) treatment increased grain number per spike, thousand grain weight, and yield by 17.5%, 4.72%, and 19.2% in 2023 and by 24.3%, 3.89%, and 23.3% in 2024, respectively. Compared with the CU100 treatment, the 80% controlled-release urea (CRU80) treatment decreased the effective spike number by 5.57% in 2023 but increased grain number per spike by 11.7% in 2023 and 7.38% in 2024. However, no significant differences in yield were observed between CU100 and CRU80 treatments in 2023 or 2024.

### 2.2. Effects of Combined Application of Controlled-Release Urea and Common Urea on Dry Matter Accumulation and Translocation

**Figure 1:**
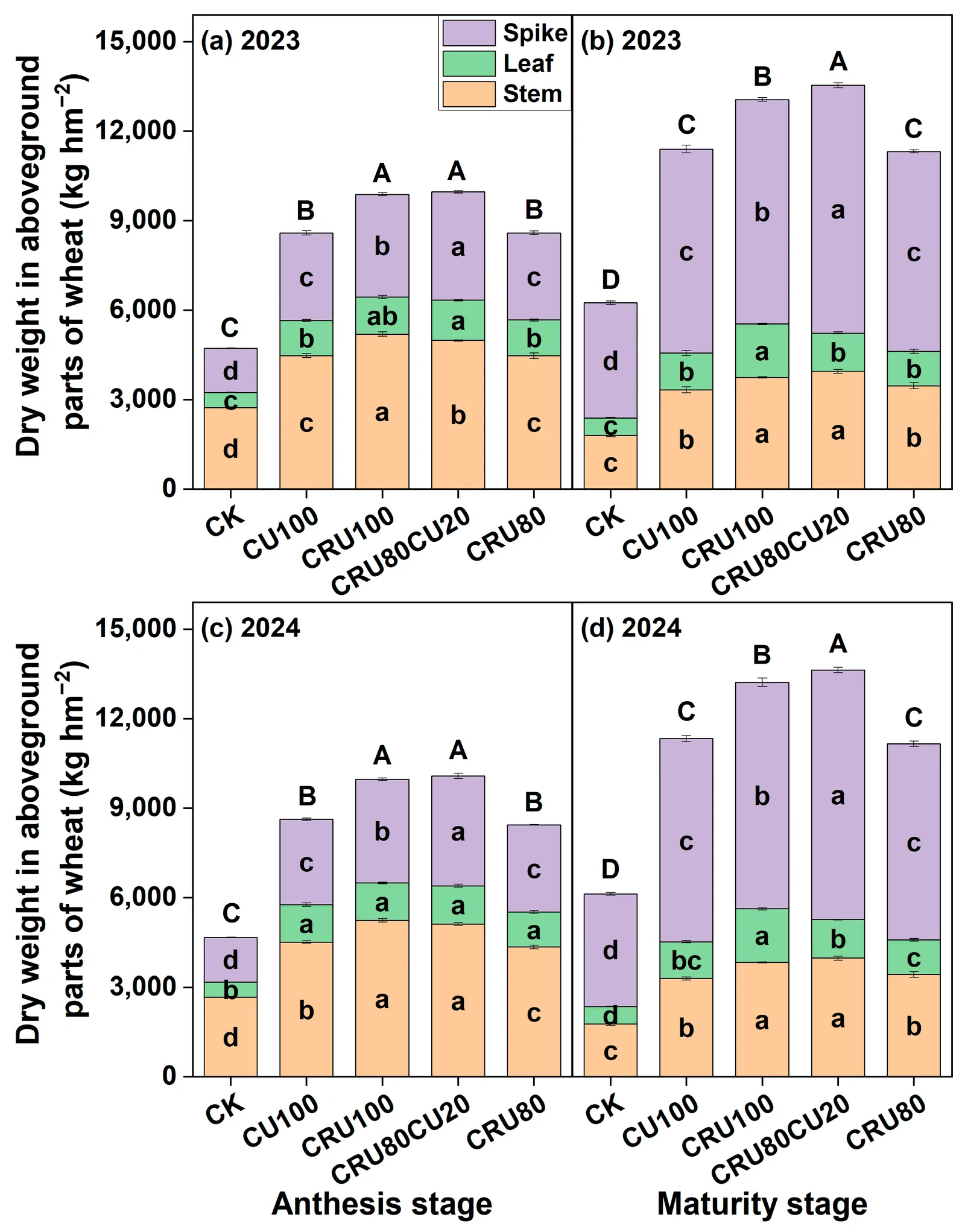
Dry matter in aboveground parts of low-gluten wheat under different nitrogen fertilizer treatments at anthesis (**a**) and maturity (**b**) stages in 2023, and at anthesis (**c**) and maturity (**d**) stages in 2024. CK: Without N fertilizer; CU100: 100% common urea; CRU100: 100% controlled-release urea; CRU80CU20: 80% controlled-release urea and 20% common urea; CRU80: 80% controlled-release urea. Means (*n* = 3) not followed by the same lowercase letter are significantly different among treatments at *p* < 0.05. Means (*n* = 3) not followed by the same uppercase letter are significantly different among treatments in the dry weight of aboveground parts of wheat within each year at *p* < 0.05.

Compared with CK treatment, different N treatments significantly increased spike, leaf, stem, and aboveground dry weight (DW) at different growth stages in 2023 and 2024 (Figure 1). Compared with the CU100 treatment, CRU100 and CRU80CU20 treatments significantly increased stem, spike, and aboveground DW at both anthesis and maturity stages in 2023 and 2024 (Figure 1). In contrast, CRU80 treatment did not significantly affect aboveground DW at either anthesis or maturity compared with CU100 (Figure 1).

**Figure 2:**
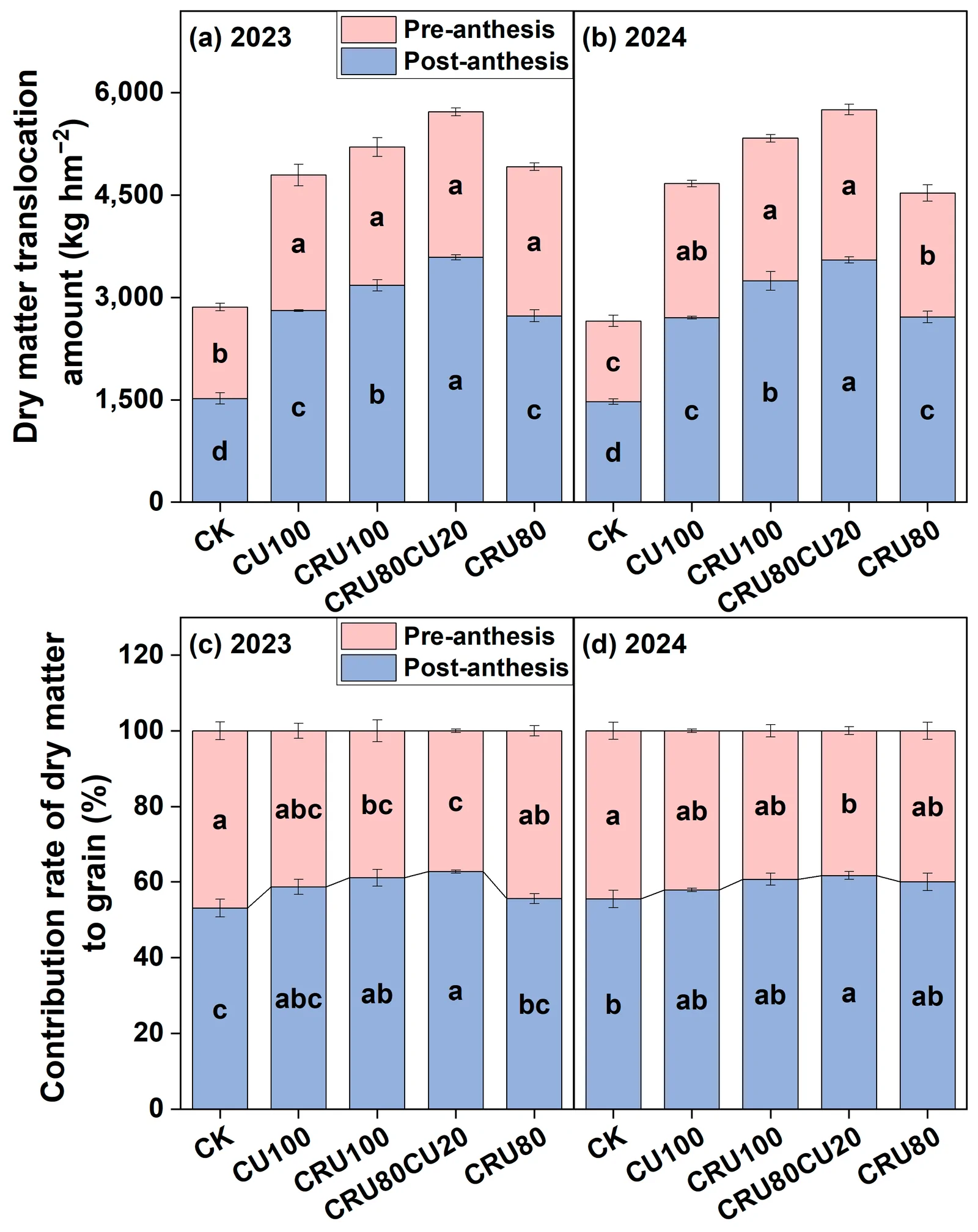
Dry matter translocation at pre-anthesis and post-anthesis in 2023 (**a**) and 2024 (**b**), and the contributions of pre-anthesis and post-anthesis dry matter to grain of low-gluten wheat in 2023 (**c**) and 2024 (**d**) under different nitrogen fertilizer treatments. CK: Without N fertilizer; CU100: 100% common urea; CRU100: 100% controlled-release urea; CRU80CU20: 80% controlled-release urea and 20% common urea; CRU80: 80% controlled-release urea. Means (*n* = 3) not followed by the same lowercase letter are significantly different among treatments within each year at *p* < 0.05.

Compared with CK treatment, different N treatments significantly increased both pre-anthesis dry matter translocation amount (Pre-DMTA) and post-anthesis dry matter accumulation (Post-DMA) (Figure 2). Compared with the CU100 treatment, the CRU100 and CRU80CU20 treatments significantly increased Post-DMA by 13.2% and 27.8% in 2023, and by 19.9% and 31.2% in 2024, respectively (Figure 2). The contribution of post-anthesis dry matter to grain in 2023 and 2024 ranged from 53.1% to 62.7% (Figure 2).

### 2.3. Effects of Combined Application of Controlled-Release Urea and Common Urea on Nitrogen Accumulation and Translocation

**Figure 3:**
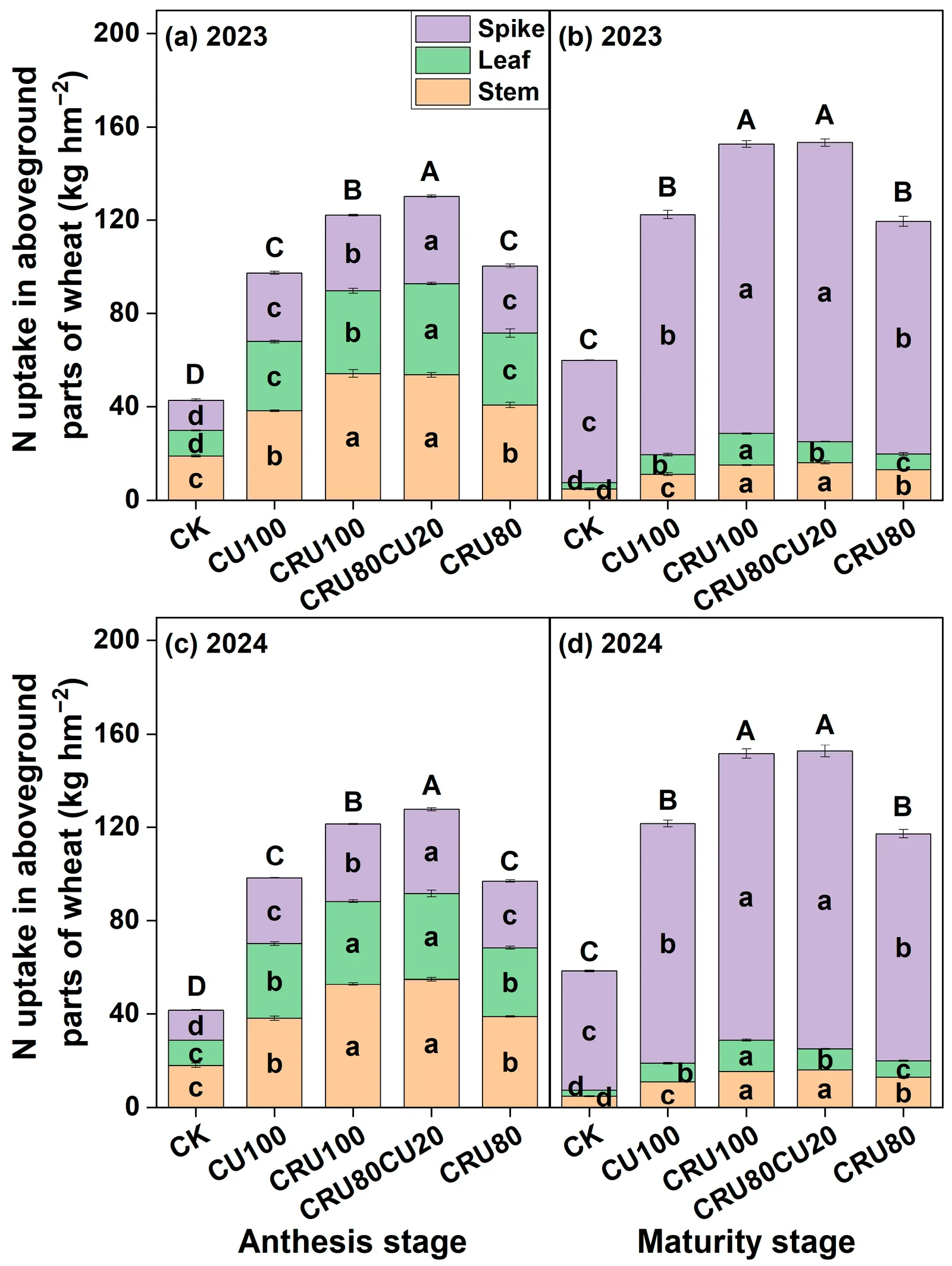
Nitrogen uptake in aboveground parts of low-gluten wheat under different nitrogen fertilizer treatments at anthesis (**a**) and maturity (**b**) stages in 2023, and at anthesis (**c**) and maturity (**d**) stages in 2024. CK: Without N fertilizer; CU100: 100% common urea; CRU100: 100% controlled-release urea; CRU80CU20: 80% controlled-release urea and 20% common urea; CRU80: 80% controlled-release urea. Means (*n* = 3) not followed by the same lowercase letter are significantly different among treatments at *p* < 0.05. Means (*n* = 3) not followed by the same uppercase letter are significantly different among treatments in N uptake of aboveground parts of wheat within each year at *p* < 0.05.

As shown in Figure 3, compared with CK treatment, all N treatments significantly increased N accumulation in spikes, leaves, stems, and aboveground parts at different stages in 2023 and 2024. Compared with CU100 treatment, CRU100 and CRU80CU20 treatments significantly increased N accumulation in stems, spikes, and aboveground parts at anthesis and maturity in 2023 and 2024. In contrast, CRU80 treatment showed no significant differences in aboveground N accumulation at anthesis or maturity compared with CU100 treatment in 2023 and 2024.

**Figure 4:**
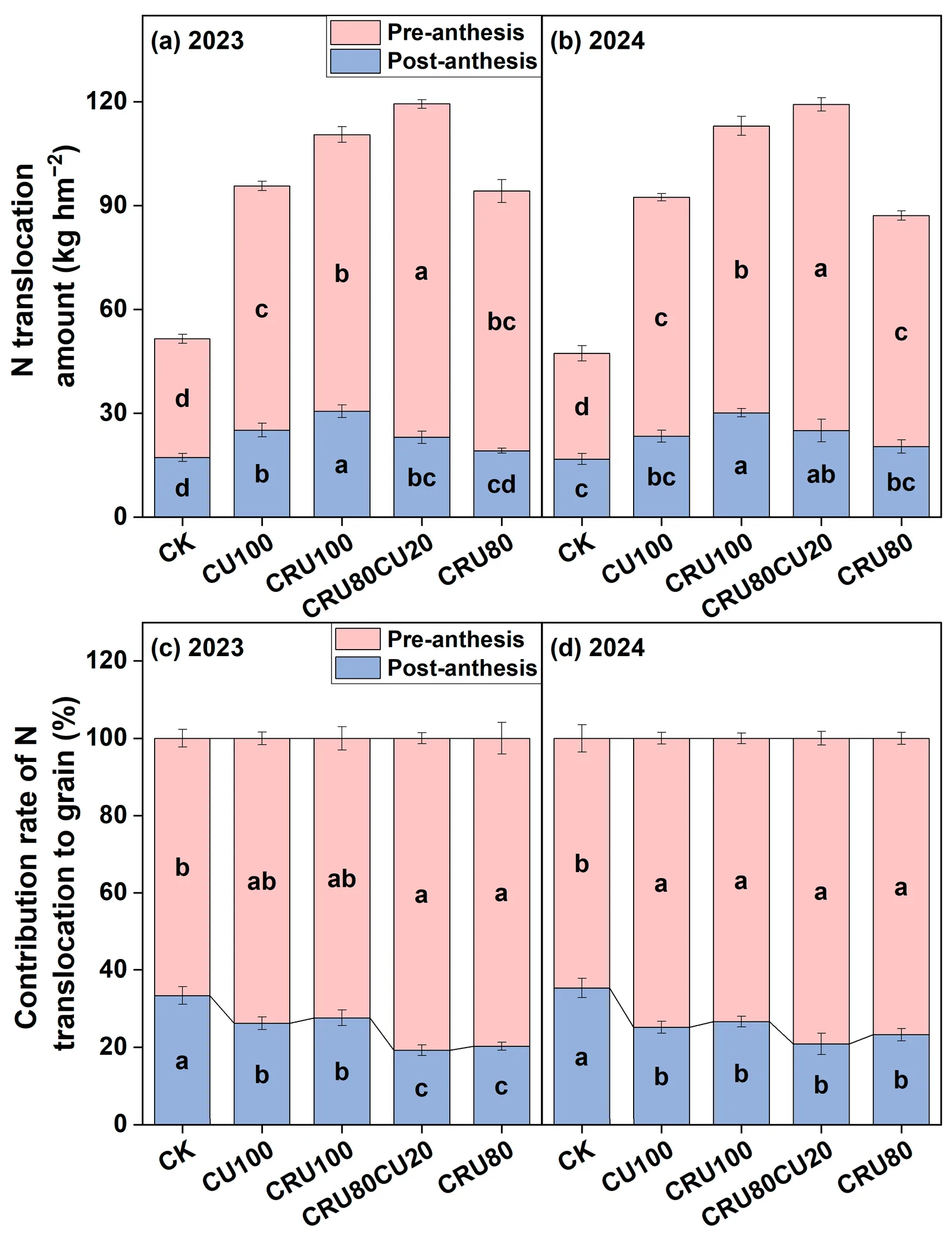
Nitrogen translocation at pre-anthesis and post-anthesis in 2023 (**a**) and 2024 (**b**), and the contributions of pre-anthesis and post-anthesis N to grain N uptake of low-gluten wheat in 2023 (**c**) and 2024 (**d**) under different nitrogen fertilizer treatments. CK: Without N fertilizer; CU100: 100% common urea; CRU100: 100% controlled-release urea; CRU80CU20: 80% controlled-release urea and 20% common urea; CRU80: 80% controlled-release urea. Means (*n* = 3) not followed by the same lowercase letter are significantly different among treatments at *p* < 0.05.

Compared with CK, different N treatments significantly increased the pre-anthesis N translocation amount (Pre-NTA) (Figure 4). Compared with the CU100 treatment, CRU100 and CRU80CU20 treatments significantly increased Pre-NTA in 2023 and 2024, whereas the CRU80 treatment significantly decreased post-anthesis N accumulation (Post-NA) in 2023 (Figure 4). The contribution rate of pre-anthesis N to grain N uptake was 64.5–80.7% (Figure 4). Compared with CK, different N treatments significantly decreased the contribution rate of post-anthesis N to grain N uptake in 2023 and 2024 (Figure 4).

### 2.4. Effects of Combined Application of Controlled-Release Urea and Common Urea on Grain Quality

**Figure 5:**
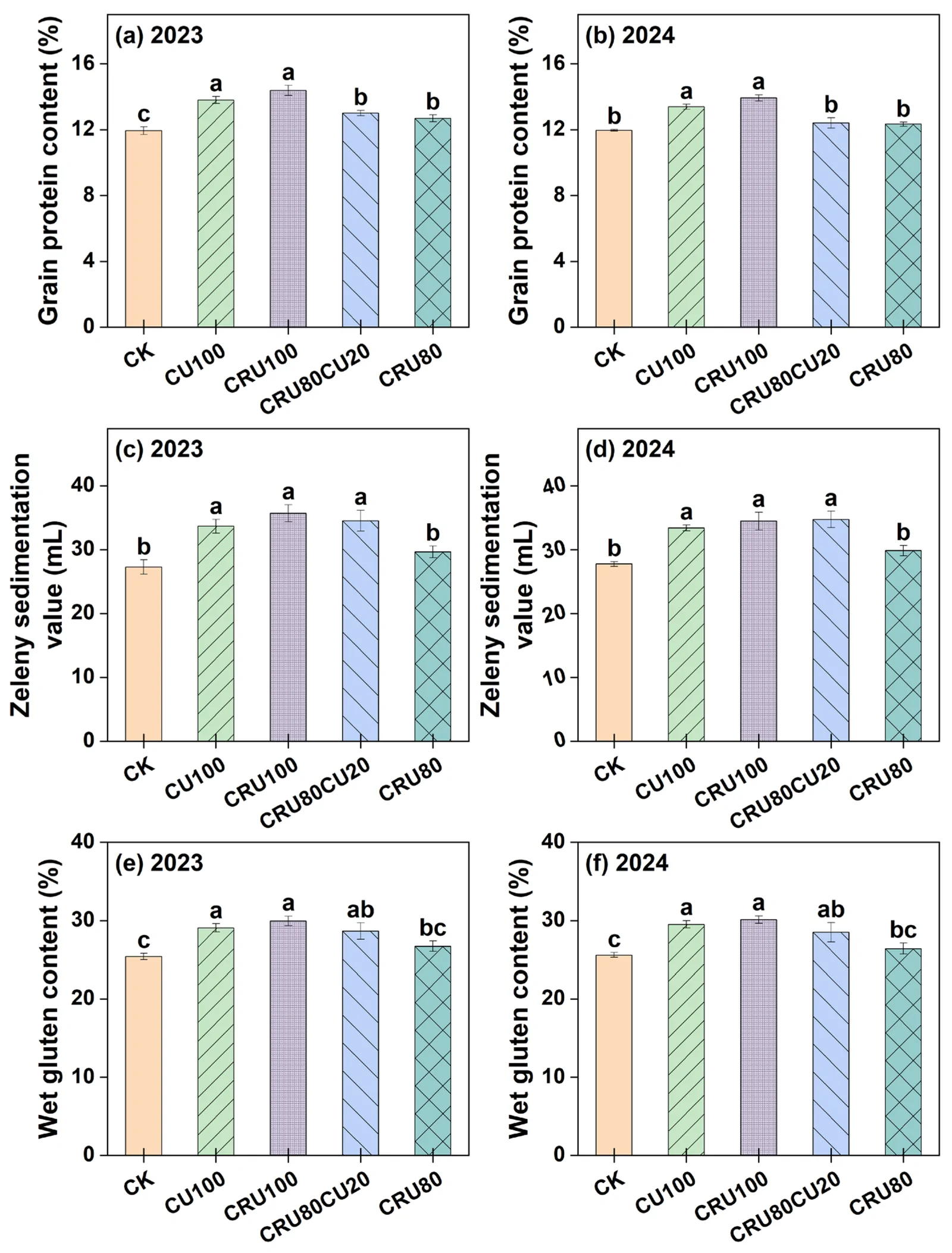
Grain protein content (**a**,**b**), Zeleny sedimentation value (**c**,**d**), and wet gluten content (**e**,**f**) of low-gluten wheat under different nitrogen fertilizer treatments in 2023 and 2024. CK: Without N fertilizer; CU100: 100% common urea; CRU100: 100% controlled-release urea; CRU80CU20: 80% controlled-release urea and 20% common urea; CRU80: 80% controlled-release urea. Means (*n* = 3) not followed by the same lowercase letter are significantly different among treatments within each year at *p* < 0.05.

There were no significant differences observed among treatments in grain moisture content, test weight, or grain hardness (Figure S1). Compared with the CU100 treatment, the CRU100 treatment did not show significant differences in grain protein content, Zeleny sedimentation value, or wet gluten content in 2023 and 2024 (Figure 5). However, compared with the CU100 treatment, the CRU80CU20 treatment significantly decreased grain protein content by 5.77% and 7.37%, and the CRU80 treatment significantly decreased grain protein content by 8.16% and 7.89%, Zeleny sedimentation value by 11.9% and 10.7%, and wet gluten content by 8.07% and 10.6% in 2023 and 2024, respectively (Figure 5).

### 2.5. Effects of Combined Application of Controlled-Release Urea and Common Urea on Soil Nitrogen and Nitrogen Use Efficiency

Compared with the CU100 treatment, the CRU100, CRU80CU20, and CRU80 treatments significantly increased NUE, agronomic efficiency of N (AEN), and partial factor productivity of N (PFPN) in both 2023 and 2024 (Table 2). The highest NUE was observed in the CRU80CU20 treatment, reaching 44.4% in 2023 and 44.9% in 2024 (Table 2). Compared with CK, different N treatments significantly increased soil total N content in both 2023 and 2024 (Table 2). Compared with the CU100 treatment, the CRU100 treatment significantly increased soil total N content in both 2023 and 2024 (Table 2). In addition, soil alkali-hydrolyzable N (SAHN) of CRU100 and CRU80CU20 treatments increased by 13.3% and 15.4% in 2023 and by 11.8% and 10.5% in 2024, compared with the CU100 treatment (Table 2).

**Figure 6:**
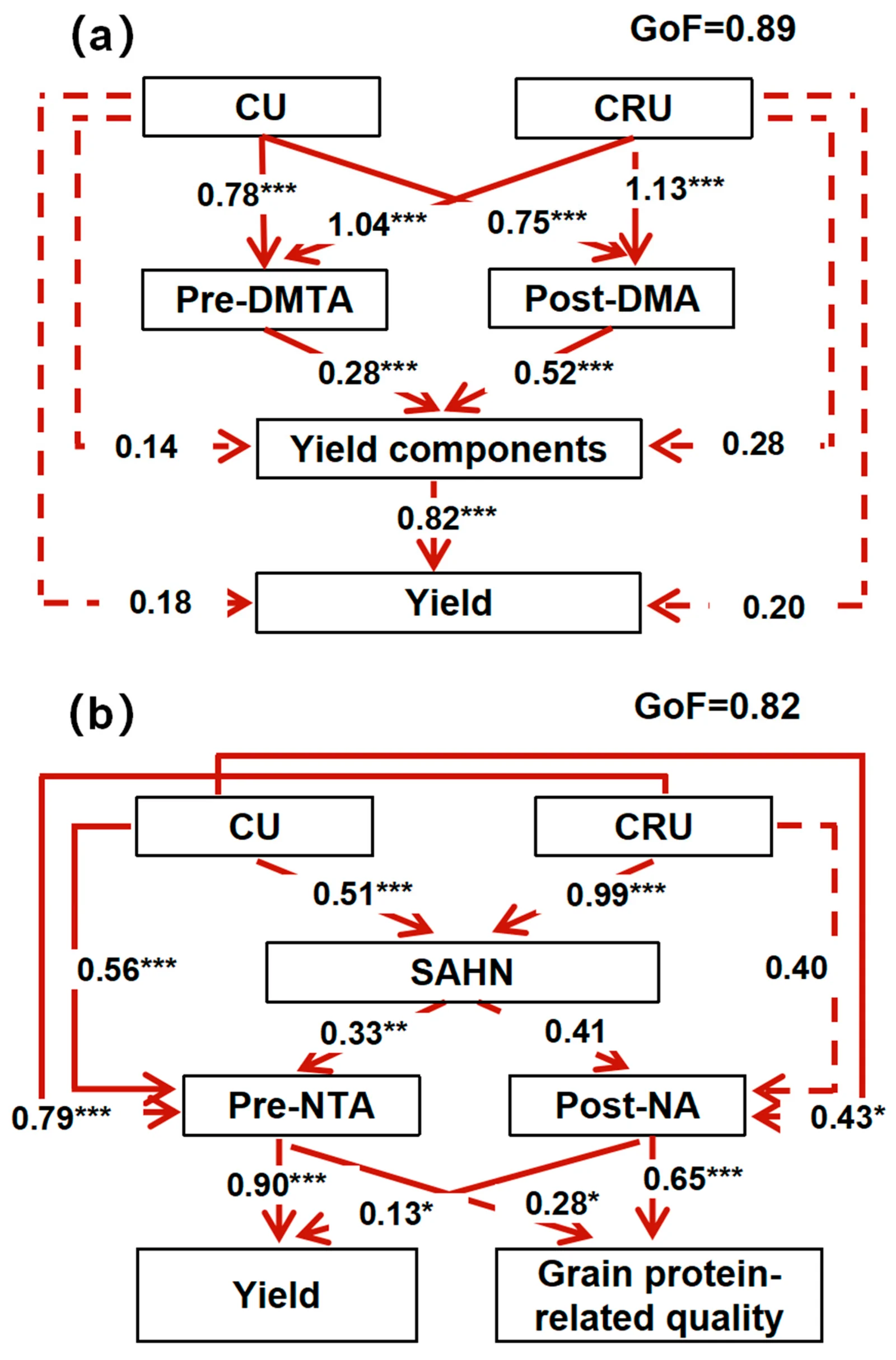
Partial least squares path modeling of the relationships among controlled-release urea, common urea, dry matter and nitrogen translocation amount, yield components, grain yield, protein-related quality indices, and soil alkali-hydrolyzable nitrogen: (**a**) dry matter translocation pathway affecting grain yield; (**b**) soil alkali-hydrolyzable nitrogen and nitrogen translocation pathway affecting grain yield and protein-related quality. CU: common urea proportion; CRU: controlled-release urea proportion; grain protein-related quality including grain protein content, Zeleny sedimentation value, and wet gluten content; Pre-DMTA: pre-anthesis dry matter translocation amount; Post-DMA: post-anthesis dry matter accumulation; Pre-NTA: pre-anthesis N translocation amount; Post-NA: post-anthesis N accumulation; SAHN: soil alkali-hydrolyzable N. GoF indicates the goodness of fit. The red lines represent positive paths; the solid and dashed lines indicate significant and non-significant paths, respectively. The values next to the solid and dashed lines are standardized path coefficients. Asterisks (*) indicate the significance of the path coefficients based on 500 bootstrap resamples (* *p* < 0.05, ** *p* < 0.01, and *** *p* < 0.001).

To further clarify the relationships among CU, CRU, soil N content, dry matter translocation, N translocation, yield, and grain protein-related quality indices, a partial least squares path modeling (PLS-PM) analysis was conducted, and the resulting path model is shown in Figure 6. The results showed that CU and CRU had significant positive paths on both Pre-DMTA and Post-DMA. Pre-DMTA and Post-DMA had significant positive paths on yield components, while yield components had a significant positive effect on yield. However, the effects of CU and CRU on yield were not significant. Both CU and CRU significantly affected SAHN. CU had significant positive paths on both Pre-NTA (0.56) and Post-NA (0.43). In contrast, CRU had a significant positive effect on Pre-NTA (0.79) but no significant effect on Post-NA. SAHN had a significant positive effect on Pre-NTA (0.33). Pre-NTA and Post-NA had significant positive paths on yield and grain protein-related quality indices. The goodness-of-fit (GoF) values are 0.89 and 0.82, respectively. However, given the relatively small sample size and standardized path coefficients greater than 1, the PLS-PM results should be considered exploratory associations rather than confirmatory evidence of causality.

## 3. Discussion

### 3.1. Effects of Controlled-Release Urea and Common Urea Combination on the Yield of Low-Gluten Wheat

Our study indicated that the CRU80CU20 treatment achieved the highest grain yield among all N treatments in 2023 and 2024, while the CRU100 treatment also significantly increased grain yield compared with the CU100 treatment. Specifically, compared with the CU100 treatment, the CRU80CU20 treatment increased grain number per spike by 17.5–24.3%, thousand grain weight by 3.89–4.72%, and yield by 19.2–23.3% over the two years (Table 1). Although the CRU80 treatment decreased the effective spike number, it partially compensated for this reduction by increasing grain number per spike by 11.7% in 2023, ultimately resulting in a grain yield comparable to that of the CU100 treatment (Table 1). These results indicated that optimizing the proportion of CRU and CU was more effective than applying CRU alone, as the CRU80CU20 treatment simultaneously improved yield components and promoted grain yield without increasing the total N supply. Previous studies have also shown that an appropriate combination of CRU and CU can improve crop productivity, potentially improving the temporal match between N availability and crop demand [8,23].

In this study, the contribution of post-anthesis dry matter to grain yield ranged from 53.1% to 62.7% over the two years (Figure 2), indicating that grain filling largely depended on the supply of assimilates after anthesis [24]. Compared with the CU100 treatment, both CRU100 and CRU80CU20 treatments significantly increased Post-DMA and aboveground dry matter in 2023 and 2024 (Figure 1 and Figure 2) and were accompanied by higher SAHN content (Table 2). The PLS-PM analysis also showed that CRU was positively associated with Pre-DMTA and Post-DMA, which were associated with yield components and grain yield (Figure 6a). Previous studies have demonstrated that maintaining adequate N availability after anthesis delays leaf senescence, prolongs canopy photosynthesis, enhances dry matter accumulation, and promotes N remobilization from vegetative organs to developing grains, ultimately improving grain yield [25,26,27,28]. These results suggested that yield increases under CRU-based treatments were primarily associated with improved dry matter accumulation and allocation. These responses may partly reflect the reported N release characteristics of CRU and the complementary N availability from CU and CRU; similar associations have also been reported in rice and maize [29,30,31]. However, this interpretation should not be taken as direct evidence that fertilizer N release matched crop demand; direct measurements of fertilizer N release and N losses in future studies would help clarify this relationship.

### 3.2. Effects of Controlled-Release Urea and Common Urea Combination on Quality of Low-Gluten Wheat

Our study showed that N management mainly affected protein-related quality indices, whereas grain moisture content, test weight, and grain hardness remained unchanged among all treatments (Figure 5 and Figure S1). Compared with the CU100 treatment, the CRU100 treatment did not significantly affect grain protein content, Zeleny sedimentation value, or wet gluten content in either 2023 or 2024 (Figure 5), suggesting that replacing CU with CRU at the same N rate was insufficient to alter the protein-related quality indices. In contrast, the CRU80CU20 treatment significantly decreased grain protein content by 5.77% and 7.37% in 2023 and 2024, respectively, while maintaining the highest grain yield (Table 1 and Figure 5). According to the quality standards for low-gluten wheat, CRU80CU20 met the protein criterion in 2024 but not in 2023, whereas its wet gluten did not meet the corresponding criteria in either year [12]. Although CRU80 further decreased the grain protein content, Zeleny sedimentation value, and wet gluten content, its yield was not significantly higher than that of CU100.

Grain quality of low-gluten wheat is sensitive to the amount and timing of N supply during grain filling, as this stage largely affects grain protein accumulation [32,33]. In the present study, pre-anthesis N translocation accounted for 64.5–80.7% of grain N uptake, indicating that remobilized N was the dominant source of grain N accumulation (Figure 4). The PLS-PM analysis further showed that both Pre-NTA and Post-NA had significant positive associations with the protein-related quality indices, including grain protein content, Zeleny sedimentation value, and wet gluten content (Figure 6b). These results suggested that the lower grain protein content observed under CRU80CU20 was associated with changes in N accumulation and translocation. Notably, although CRU80CU20 significantly decreased grain protein content, CRU100 did not show a similar effect, suggesting that a reduction in the CU proportion alone may not explain the lower protein content. A possible explanation is that the appropriate CU proportion in CRU80CU20 might alter the timing of N availability and Post-NA, thereby potentially limiting excessive protein accumulation.

CU is rapidly hydrolyzed and may result in high N availability during early growth, followed by lower N availability during grain filling when the risk of N losses is substantial [34,35]. Compared with the CU100 treatment, CRU100 and CRU80CU20 treatments significantly increased SAHN at the maturity stage in 2023 and 2024 (Table 2). However, PLS-PM indicated that CRU was positively associated with Pre-NTA but not with Post-NA, whereas SAHN was significantly and positively associated with Pre-NTA (Figure 6b). These results suggested that the yield differences among CRU-based treatments might be related to higher SAHN and enhanced Pre-NTA rather than increased Post-NA alone. Compared with the CRU100 treatment, the CRU80CU20 treatment combined readily available N from CU with the gradual release characteristics of CRU, thereby supporting biomass and yield formation while limiting excessive grain protein accumulation. However, N fertilizer release and soil mineral N dynamics were not measured, and their potential mechanisms need further investigation.

### 3.3. Effect of Controlled-Release Urea and Common Urea Combination on Nitrogen Use Efficiency and Soil N Content

Our study showed that all CRU treatments significantly increased NUE, AEN, and PFPN, with increases of 16.3–49.5%, 16.8–54.3%, and 8.77–28.5%, respectively, compared with the CU100 treatment (Table 2). The CRU80CU20 treatment achieved the highest NUE and grain yield in both 2023 and 2024. These findings suggested that combining CU with CRU might reflect complementary patterns of N availability, as previous studies have indicated that the rapid hydrolysis of CU might increase early N losses and limit N availability during grain filling, whereas the gradual N release of CRU could maintain N availability in cereals during reproductive growth and support N uptake and translocation [34,36,37,38]. This complementary N supply pattern may have promoted N uptake, dry matter accumulation, and N translocation, thereby contributing to the high yield and NUE observed under CRU80CU20. However, because CRU80 received 20% less N than CU100, CRU100, and CRU80CU20, its responses may reflect the combined effects of N rate and fertilizer formulation and should therefore be interpreted as indicating the potential for N reduction. Accordingly, fertilizer effects were interpreted primarily from comparisons among CU100, CRU100, and CRU80CU20, which received the same total N rate.

Compared with the CU100 treatment, CRU100 and CRU80CU20 treatments significantly increased SAHN content by 10.5–15.4% at maturity in 2023 and 2024 (Table 2). The PLS-PM analysis further showed that both CU and CRU were positively associated with SAHN, and SAHN had a significant positive association with Pre-NTA (Figure 6b). These results suggested that soil N availability was associated with N accumulation and redistribution before anthesis, which may have contributed to yield formation. Moreover, SAHN content was measured only at harvest; these relationships could not demonstrate that CRU maintained greater soil N availability throughout the growing season. In addition, CU had significant positive paths to both Pre-NTA and Post-NA, whereas CRU had a significant path to Pre-NTA but not to Post-NA (Figure 6b). This suggested that the effects of CRU-based fertilization on NUE were primarily associated with pre-anthesis N uptake and translocation rather than with increased Post-NA. Therefore, an appropriate fertilizer formulation might support NUE and crop yield by maintaining an adequate soil N supply, rather than increasing residual soil N [39].

The CRU80CU20 treatment achieved the highest grain yield with high NUE and lower grain protein content than the CU100 treatment. At a wheat price of approximately CNY 2.4 kg^−1^, the N fertilizer costs were approximately CNY 1096 hm^−2^ for CRU80CU20 and CNY 913 hm^−2^ for CU100. Thus, the additional fertilizer cost was approximately CNY 183 hm^−2^, whereas the observed yield increases generated additional gross grain revenue of approximately CNY 2215 hm^−2^ in 2023 and CNY 2606 hm^−2^ in 2024. After subtracting the additional N fertilizer cost, the corresponding incremental returns were approximately CNY 2032 and 2423 hm^−2^, respectively. This preliminary, partial economic assessment did not establish overall profitability because labor, machinery, quality premiums, and fertilizer-price fluctuations were not considered.

Compared with the CRU100 treatment, the CRU80CU20 treatment resulted in higher grain yield and lower grain protein content. This difference may reflect the potentially complementary N availability provided by readily available N from CU and more sustained N from CRU. Consequently, CRU80CU20 maintained greater Pre-DMTA and Post-DMA, while the PLS-PM analysis indicated associations of Pre-NTA and Post-NA with yield and protein-related quality indices (Figure 6). However, fertilizer release, environmental N losses, and residual effects on subsequent crops were not measured. Therefore, improved long-term soil fertility and environmental sustainability were not established. Moreover, the experiment involved one cultivar at one location, with fixed treatment positions over two years. Broader recommendations require multi-cultivar, multi-location, and long-term trials incorporating comprehensive economic and environmental assessments. Molecular approaches may further clarify the mechanisms linking N transport, carbon allocation, and grain quality formation in low-gluten wheat.

## 4. Materials and Methods

### 4.1. Experimental Design

**Figure 7:**
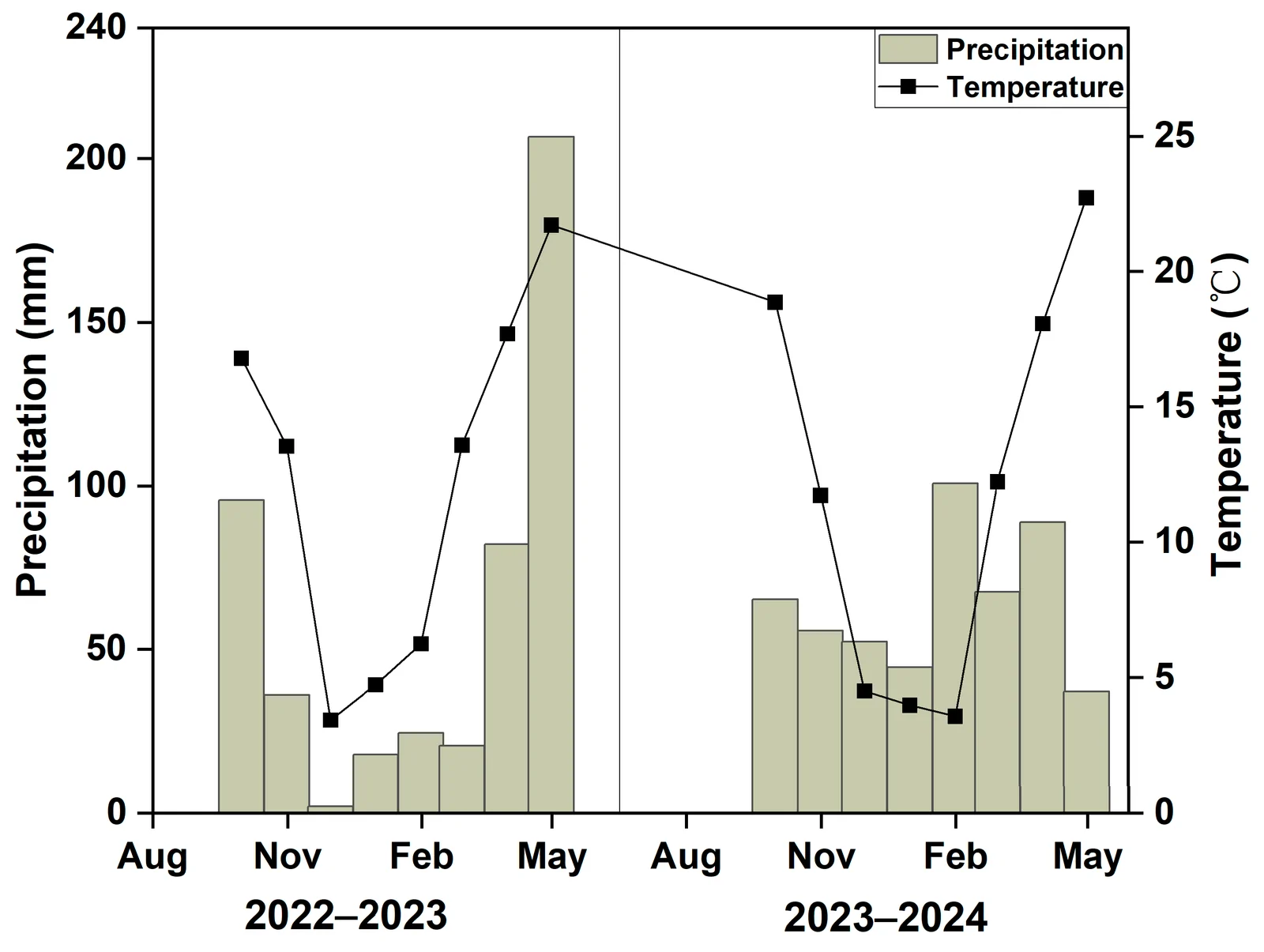
Monthly precipitation and mean air temperature during the 2022–2023 and 2023–2024 wheat-growing seasons at the experimental site. Bars indicate monthly precipitation, and the line indicates monthly mean temperature.

This field experiment was conducted in Xixian County, Henan Province, China (32.15° N, 114.09° E). This site is situated in a typical agricultural area of the Huang-Huai Plain, characterized by suitable climatic conditions and soil properties for wheat production. Wheat was grown without irrigation in either experimental season, relying entirely on precipitation, with temperature and precipitation for growing seasons presented in Figure 7. The previous crop was soybean, and all soybean straw was chopped and returned to the field. The soil texture is clay loam, and physicochemical properties included soil pH of 7.61, organic matter content of 19.3 g kg^−1^, total N content of 0.70 g kg^−1^, alkali-hydrolyzable N (SAHN) content of 60.4 mg kg^−1^, available phosphorus content of 8.15 mg kg^−1^, and available potassium content of 120 mg kg^−1^.

This experiment consisted of five treatments with three replicates: without N fertilizer (CK), 100% common urea (CU100, 210 kg N hm^−2^), 100% controlled-release urea (CRU100, 210 kg N hm^−2^), 80% controlled-release urea and 20% common urea (CRU80CU20, 210 kg N hm^−2^), and 80% controlled-release urea (CRU80, 168 kg N hm^−2^). The rate of 210 kg N hm^−2^ represented the conventional N rate used on the experimental farm. The CRU80 treatment was conducted to evaluate sole CRU application under a 20% reduction in total N input. Both CU and CRU contained 46% N (Xinlianxin, Henan, China), and both fertilizers were applied once as basal fertilizers one week before sowing according to local practice. For the CRU80CU20 treatment, the weighed CU and CRU were thoroughly mixed at an 80:20 ratio before application. N fertilizer treatments were broadcast uniformly and then incorporated into the 0–30 cm soil layer by tillage. All treatments received the same tillage method and incorporation depth. Phosphorus and potassium fertilizers were applied uniformly to all treatments at rates equivalent to 126 kg P hm^−2^ and 105 kg K hm^−2^, respectively. This field experiment was arranged in a randomized complete block design in the first year. Each plot area was 20 m^2^ (4 m × 5 m) with a 30 cm gap between plots, and furrows were made. The same plots and treatment positions were maintained in the second season, and the same fertilizer treatments were applied. Yangmai 13 was selected because it is a representative low-gluten cultivar and one of the locally cultivated wheat cultivars. Wheat was sown on 19 October 2022 and 21 October 2023.

### 4.2. Plant and Soil Physicochemical Property Collection and Analysis

Plant samples were collected at anthesis (April 20) and maturity (May 23) stages in 2023 and at anthesis (April 21) and maturity (May 24) stages in 2024. At each stage, 10 plants were randomly sampled from the interior of each plot after excluding border rows. At anthesis, plants were separated into stems, leaves, and spikes. At maturity, plants were separated into stems, leaves, glumes, and grain. Plant height and yield components, including grain number per spike, were based on 10 plants per plot. A uniform area (10 m^2^) in each plot was harvested at maturity to determine grain yield. Samples from each stage were oven-dried at 105 °C for 30 min and then at 70 °C to constant weight. Dry weight (DW) of each organ was measured, and plant samples were then ground and stored. The dried plant samples were digested with concentrated H_2_SO_4_-H_2_O_2_, and then, total N concentrations were determined by the Kjeldahl method [40]. Grain quality was assessed after harvest. The grain moisture content, protein content, hardness, Zeleny sedimentation value, and wet gluten content of wheat were determined using a FOSS near-infrared grain analyzer (Infratec^TM^ 1241, FOSS, Hillerød, Denmark) [41]. Test weight of grain was measured using an HGT-1000A (Huake Instrument Equipment Co., Ltd., Shanghai, China) grain bulk density meter. Soil samples were collected from each plot at maturity. After air-drying, the soil was ground, sieved, and stored. Soil total N was determined by the Kjeldahl method, and SAHN was determined by the alkaline diffusion method [42].

### 4.3. Calculation and Statistical Analyses

Data normality and homogeneity of variance were tested using the Shapiro–Wilk and Levene’s tests before analysis of variance (ANOVA); the analyzed variables satisfied these assumptions. For each year, different treatment effects were evaluated using one-way ANOVA, followed by Duncan’s multiple range test at *p* < 0.05. All statistical analyses were conducted using SPSS 22.0, and figures were generated with Origin 2022. PLS-PM was performed using the “plspm” package in R (version 4.5.3). The analysis included 30 plot-year observations (5 treatments × 3 replicates × 2 years). CU proportion and CRU proportion were coded as the percentages of the N rate supplied by each fertilizer (CK: 0 and 0; CU100: 100 and 0; CRU100: 0 and 100; CRU80CU20: 20 and 80; CRU80: 0 and 80, respectively). Path significance was evaluated using 500 bootstrap resamples, and model fit was evaluated using the goodness-of-fit (GoF) index. The results are presented as means ± standard errors.

The calculation formulas for each index are as follows [43]:

Pre-anthesis dry matter translocation amount (Pre-DMTA, kg hm^−2^) = dry matter at anthesis − dry matter at maturity (excluding grains)

Post-anthesis dry matter accumulation (Post-DMA, kg hm^−2^) = grain dry weight at maturity − Pre-DMTA

Contribution rate of pre-anthesis dry matter to grain (%) = (Pre-DMTA/grain dry weight at maturity) × 100

Contribution rate of post-anthesis dry matter to grain (%) = (Post-DMA/grain dry weight at maturity) × 100

Pre-anthesis N translocation amount (Pre-NTA, kg hm^−2^) = N uptake at anthesis − N uptake at maturity (excluding grains)

Post-anthesis N accumulation (Post-NA, kg hm^−2^) = plant N uptake at maturity − plant N uptake at anthesis

Contribution rate of pre-anthesis N to grain N (%) = (Pre-NTA/grain N uptake at maturity) × 100

Contribution rate of post-anthesis N to grain N (%) = (Post-NA/grain N uptake at maturity) × 100

N use efficiency (NUE, %) = (N accumulation in plants in fertilized plot − N accumulation in plants in CK treatment)/N application rate × 100

Agronomic efficiency of N (AEN, kg kg^−1^) = (grain yield in fertilized plot − grain yield in CK treatment)/N application rate

Partial factor productivity of N (PFPN, kg kg^−1^) = grain yield in fertilized plot/N application rate

## 5. Conclusions

This study demonstrated that both CRU100 and CRU80CU20 treatments enhanced grain yield primarily by increasing grain number per spike, promoting dry matter accumulation, and improving N uptake and translocation compared with the CU100 treatment. The PLS-PM analysis further revealed that Post-DMA was strongly associated with yield, whereas both Pre-NTA and Post-NA were positively associated with the protein-related quality indices. Although CRU100 improved grain yield and NUE, it did not significantly affect grain protein content. In contrast, CRU80CU20 significantly decreased grain protein content while maintaining high grain yield compared with the CU100 treatment. Moreover, CRU100 and CRU80CU20 treatments increased SAHN at maturity, while all CRU treatments significantly enhanced NUE, AEN, and PFPN. Among all treatments, the CRU80CU20 treatment provided a favorable combination of grain yield, grain protein content, NUE, and soil N content, although it did not consistently meet all low-gluten wheat quality criteria across the two growing seasons. Therefore, the combined application of CRU and CU may provide a practical N management strategy for coordinating grain yield, quality, and NUE in low-gluten wheat. However, broader agronomic recommendations require validation across cultivars and locations, as well as assessments of economic performance, environmental N losses, and long-term soil residual effects.

## Figures and Tables

**Table 1:** Effects of different nitrogen fertilizer treatments on yield and yield components of low-gluten wheat in 2023 and 2024.

Year	Treatment	Plant Height (cm)	Effective Spike Number (×10^4^ hm^−2^)	Grain Number per Spike	Thousand Grain Weight (g)	Yield (kg hm^−2^)
2023	CK	53.9 ± 1.12 b	303 ± 6.30 c	28.7 ± 0.67 e	35.6 ± 0.84 c	2858 ± 35.0 d
	CU100	65.9 ± 0.44 a	413 ± 3.45 a	34.3 ± 0.88 d	38.1 ± 0.09 b	4796 ± 154 c
	CRU100	67.4 ± 1.65 a	417 ± 7.84 a	36.3 ± 0.33 c	39.1 ± 0.47 ab	5204 ± 68.0 b
	CRU80CU20	68.1 ± 0.71 a	423 ± 4.87 a	40.3 ± 0.33 a	39.9 ± 0.24 a	5719 ± 96.9 a
	CRU80	68.6 ± 0.46 a	390 ± 3.85 b	38.3 ± 0.67 b	39.1 ± 0.48 ab	4915 ± 60.7 bc
2024	CK	52.6 ± 0.52 b	300 ± 3.32 c	29.5 ± 0.55 e	35.3 ± 0.65 c	2654 ± 59.0 d
	CU100	66.9 ± 2.13 a	404 ± 6.69 b	32.5 ± 0.69 d	38.6 ± 0.34 b	4668 ± 62.1 c
	CRU100	65.3 ± 0.29 a	413 ± 3.38 ab	38.5 ± 0.87 b	39.6 ± 0.28 ab	5334 ± 94.6 b
	CRU80CU20	66.5 ± 1.61 a	424 ± 2.19 a	40.4 ± 0.03 a	40.1 ± 0.08 a	5754 ± 55.3 a
	CRU80	65.1 ± 0.76 a	395 ± 4.36 b	34.9 ± 0.20 c	38.5 ± 0.62 b	4529 ± 71.3 c

CK: Without N fertilizer; CU100: 100% common urea; CRU100: 100% controlled-release urea; CRU80CU20: 80% controlled-release urea and 20% common urea; CRU80: 80% controlled-release urea. Means (*n* = 3) not followed by the same lowercase letter are significantly different among treatments within each year at *p* < 0.05.

**Table 2:** Effects of different nitrogen fertilizer treatments on N use efficiency and soil N content of low-gluten wheat at the maturity stage in 2023 and 2024.

Year	Treatment	N Use Efficiency (%)	Agronomic Efficiency of N (kg kg^−1^)	Partial Factor Productivity of N (kg kg^−1^)	Soil Total N (g kg^−1^)	Soil Alkali-Hydrolyzable N (mg kg^−1^)
2023	CK	/	/	/	0.61 ± 0.00 d	49.0 ± 0.93 b
	CU100	29.7 ± 1.06 c	9.23 ± 0.88 c	22.8 ± 0.73 d	0.69 ± 0.01 bc	51.9 ± 0.95 b
	CRU100	44.2 ± 0.63 a	11.2 ± 0.46 b	24.8 ± 0.32 c	0.73 ± 0.00 a	58.8 ± 0.51 a
	CRU80CU20	44.4 ± 0.82 a	13.6 ± 0.31 a	27.2 ± 0.46 b	0.71 ± 0.00 ab	59.9 ± 0.90 a
	CRU80	35.5 ± 1.65 b	12.2 ± 0.55 ab	29.3 ± 0.36 a	0.67 ± 0.02 c	51.7 ± 1.79 b
2024	CK	/	/	/	0.60 ± 0.00 d	46.7 ± 1.50 c
	CU100	30.1 ± 0.62 c	9.59 ± 0.22 d	22.2 ± 0.30 c	0.72 ± 0.00 b	55.0 ± 1.26 b
	CRU100	44.3 ± 1.30 a	12.8 ± 0.32 b	25.4 ± 0.45 b	0.74 ± 0.00 a	61.5 ± 0.57 a
	CRU80CU20	44.9 ± 1.55 a	14.8 ± 0.43 a	27.4 ± 0.26 a	0.72 ± 0.00 b	60.8 ± 0.81 a
	CRU80	35.0 ± 1.51 b	11.2 ± 0.74 c	27.0 ± 0.42 a	0.70 ± 0.01 c	54.0 ± 1.52 b

CK: Without N fertilizer; CU100: 100% common urea; CRU100: 100% controlled-release urea; CRU80CU20: 80% controlled-release urea and 20% common urea; CRU80: 80% controlled-release urea. Means (*n* = 3) not followed by the same lowercase letter are significantly different among treatments within each year at *p* < 0.05.

## Data Availability

The original contributions presented in this study are included in the article. Further inquiries can be directed to the corresponding author.

## Supplementary Materials

The following supporting information can be downloaded at https://www.mdpi.com/article/10.3390/plants15172728/s1, Figure S1: Effects of different nitrogen fertilizer treatments on grain moisture content, test weight, and grain hardness of low-gluten wheat in 2023 and 2024. CK: Without N fertilizer; CU100: 100% common urea; CRU100: 100% controlled-release urea; CRU80CU20: 80% controlled-release urea and 20% common urea; CRU80: 80% controlled-release urea. Means (*n* = 3) not followed by the same lowercase letter are significantly different among treatments within each year at *p* < 0.05.
