# Supplementary material for: Combined Application of Controlled-Release Urea and Common Urea Improves Yield and Nitrogen Use Efficiency in Low-Gluten Wheat
Source: Plants (Basel). 2026 Sep 6;15(17):2728. doi: 10.3390/plants15172728 (PMC13567705; doi:10.3390/plants15172728)
Supplement: Supplementary file 1 [file plants-15-02728-s001.zip › plants-4503801-supplementary.pdf]

Supplementary material

Combined application of controlled-release urea and common urea improves yield and nitrogen use efficiency in low-gluten wheat

Di Li [1,†], Guanghui Du [2,†], Liyang Chen [2], Jianbo Yang [1], Guobing Wang [1], Yuhu Lv [2], Yao Liu [2,*], and Xun Li [3,*]

[1] Institute of Geographical Sciences, Henan Academy of Sciences, Zhengzhou 450052, China
[2] Xinyang Academy of Agricultural Sciences, Xinyang, 464001, China
[3] State Key Laboratory of Soil and Sustainable Agriculture, Institute of Soil Science, Chinese Academy of Sciences, Nanjing, 211135, China

* Correspondence: liuyao19930103@163.com (Y. L.), xli@issas.ac.cn (X. L.)
† These authors contributed equally to this work.

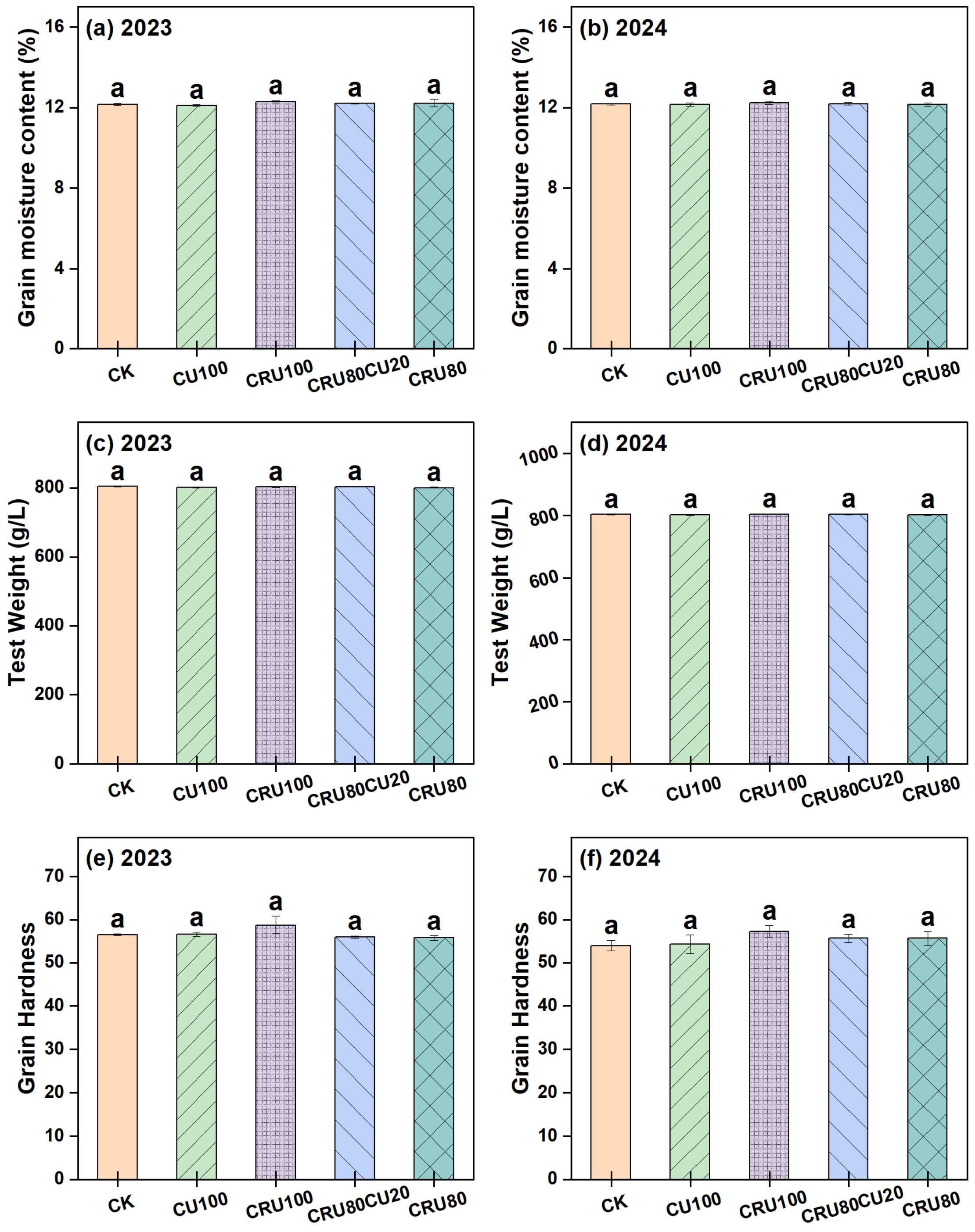

Figure S1. Effects of different nitrogen fertilizer treatments on grain moisture content, test weight, and grain hardness of low-gluten wheat in 2023 and 2024. CK: without N fertilizer; CU100: 100% common urea; CRU100: 100% controlled-release urea; CRU80CU20: 80% controlled-release urea and 20% common urea; CRU80: 80% controlled-release urea. Means (n = 3) not followed by the same lowercase letter are significantly different among treatments within each year at $P < 0.05$.
